# Stoichiometry and Change of the mRNA Closed-Loop Factors as Translating Ribosomes Transit from Initiation to Elongation

**DOI:** 10.1371/journal.pone.0150616

**Published:** 2016-03-08

**Authors:** Xin Wang, Wen Xi, Shaun Toomey, Yueh-Chin Chiang, Jiri Hasek, Thomas M. Laue, Clyde L. Denis

**Affiliations:** 1 Department of Molecular, Cellular, and Biomedical Sciences, University of New Hampshire, Durham, NH, 03824, United States of America; 2 Laboratory of Cell Reproduction, Institute of Microbiology of ASCR, Prague, Videnska 1083, Czech Republic; University of British Columbia, CANADA

## Abstract

Protein synthesis is a highly efficient process and is under exacting control. Yet, the actual abundance of translation factors present in translating complexes and how these abundances change during the transit of a ribosome across an mRNA remains unknown. Using analytical ultracentrifugation with fluorescent detection we have determined the stoichiometry of the closed-loop translation factors for translating ribosomes. A variety of pools of translating polysomes and monosomes were identified, each containing different abundances of the closed-loop factors eIF4E, eIF4G, and PAB1 and that of the translational repressor, SBP1. We establish that closed-loop factors eIF4E/eIF4G dissociated both as ribosomes transited polyadenylated mRNA from initiation to elongation and as translation changed from the polysomal to monosomal state prior to cessation of translation. eIF4G was found to particularly dissociate from polyadenylated mRNA as polysomes moved to the monosomal state, suggesting an active role for translational repressors in this process. Consistent with this suggestion, translating complexes generally did not simultaneously contain eIF4E/eIF4G and SBP1, implying mutual exclusivity in such complexes. For substantially deadenylated mRNA, however, a second type of closed-loop structure was identified that contained just eIF4E and eIF4G. More than one eIF4G molecule per polysome appeared to be present in these complexes, supporting the importance of eIF4G interactions with the mRNA independent of PAB1. These latter closed-loop structures, which were particularly stable in polysomes, may be playing specific roles in both normal and disease states for specific mRNA that are deadenylated and/or lacking PAB1. These analyses establish a dynamic snapshot of molecular abundance changes during ribosomal transit across an mRNA in what are likely to be critical targets of regulation.

## Introduction

The regulation of protein synthesis is central to the formation of proteins in all organisms. Much of this control involves changes in abundances and activities of a variety of proteins associated with the translating ribosome. The current model for translation indicates that in protein synthesis the mRNA forms a putative closed-loop structure in which eIF4E, the 5’ mRNA cap binding protein, binds eIF4G, which in turn binds the poly(A)-binding protein (PAB1) that is bound to the 3’ poly(A) tail of mRNA [1–3]. This structure would link the 5’ end of the mRNA to the 3’ end. The resultant complex interacts with the 43S complex (40S small ribosomal subunit, translation initiation factors eIF2, -3, -5, and -1 and the charged methionine tRNA) to form the 48S complex. This 48S complex then scans for the initiation codon and brings in the 60S large ribosomal subunit to form the 80S ribosome bound to the mRNA for the start of protein synthesis [4]. Translation termination involves eRF1 recognition of the stop codon that in consort with other proteins ends protein synthesis [5,6].

Many of the studies leading to this model of translation have relied upon in vitro analyses, and they and in vivo experiments have not clearly indicated the absolute abundances of the closed-loop factors in the translating ribosome or how their abundances change during translation. For example, eRF1 has been shown to associate early with the mRNA during translation initiation based on in vitro experiments, but at what abundance it associates is not known [7]. Also, several studies have suggested that eIF4E/eIF4G can form a closed-loop structure in the absence of PAB1, but the prevalence of this type of structure as compared to the canonical closed-loop structure containing all three components has not been defined [8–10].

A quantitative determination of the components present at different stages of translation is required to obtain a fuller understanding of this process. Current biochemical and molecular biological techniques such as mass spectrometry or sucrose gradient analysis and similar chromatographic techniques give only a very crude characterization of the stoichiometry within translation complexes. For instance, mass spectrometric procedures can identify components that are present in complexes but do not readily inform about the size of the complex analyzed or the quantitation for the components of the complex. Sucrose gradient analysis and similar chromatographic techniques identify sizes, but give only rough information about component numbers (usually relying on the time-consuming and imprecise Western analysis).

The recent demonstration that analytical ultracentrifugation with fluorescent detection (AU-FDS) can rapidly and precisely identify sizes, components, and changes in composition of multiple protein complexes [11,12] indicates that AU-FDS can produce information presently either unavailable or difficult to obtain. We have consequently expanded the use of AU-FDS to determine the absolute abundances of proteins within protein synthesis complexes using the translating ribosome as our model system. The basic technique utilizes our previous AU-FDS identification of the translating ribosomes following a one-step affinity purification step using a Flag-tagged component of the protein synthesis machinery. These complexes consist of 40S and 60S ribosomal subunits, the translational initiation factors eIF4E, eIF4G, and PAB1 [11] and at least five other proteins: eRF1 (translation termination), SBP1 (translational repression) [13], and the general mRNA binding proteins SLF1, SSD1, and PUB1 [12]. AU-FDS combined with the one-step purification of translating complexes also specifically offers an opportunity to study the 77S monosomal translating complex. Previously, the co-migration of the 80S free ribosome with the 77S monosomal translating complex following sucrose gradient centrifugation studies has not allowed the detection of the 77S monosomal translating complex, although studies have suggested that it makes up about 5% of the total translating ribosomal pool [14].

We have consequently determined the absolute concentration of components of the 77S monosomal and polysomal complexes during initiation and elongation. Our AU-FDS results indicate that translating ribosomes undergo a dynamic shift in factors such as eIF4E, eIF4G, and SBP1 during translation: polysomal complexes stabilize the closed-loop structure, whereas monosomal translating complexes display significant loss of eIF4E, specific dissociation of eIF4G, and deadenylation. Translation complexes appeared to contain two types of closed-loop structures, those carrying eIF4E/eIF4G/PAB1 and those containing just eIF4E/eIF4G, confirming the importance of eIF4G interactions with the mRNA. The identification of clearly differentiable translating complexes suggests that translation is not monolithic and there exist a sizeable number of ribosomal complexes with diverse quantitative variations in the closed-loop components. These various pools imply complex regulation and specific mRNA-dependent effects.

## Results

### Stoichiometric analysis of the components in the polysomal and monosomal translational complexes

A stoichiometric analysis of the monosomal 77S and polysomal translating complexes was undertaken to provide a quantitative view of the translation machinery in terms of each of the key closed-loop structure components (eIF4E, eIF4G, and PAB1), eRF1, and SBP1 as ribosomes transit the mRNA. Starting with the 77S monosomal translating complex, in order to determine the absolute abundance of each component in this translating complex, we combined two kinds of AUC analyses. First, AU-FDS analysis monitored the fluorescent intensity of a GFP-tagged protein in the 77S complex and, consequently, informed as to the abundance of the GFP-tagged protein in the complex. Second, AU-absorption analysis was conducted at 230 nm, taking advantage of absorption of the peptide backbone at this wavelength. AU-A_230_ analysis quantitatively determined how much material, both total protein and RNA (about 3.2-fold better detection of protein than RNA at this wavelength), was actually analyzed in the AU-FDS analysis. AUC analysis, because of its much higher sensitivity and increased resolution as compared to Western blotting assays, allowed us to calculate the exact and absolute abundance of each GFP-tagged protein relative to the total amount of material within the 77S complex.

To conduct these experiments, cycloheximide was added to cells prior to cell lysis to ensure that the 77S monosomal translating complex remained attached to the mRNA during purification and analysis. Previous analyses indicated that about half of the translating ribosomes run-off the mRNA in the absence of cycloheximide [11]. To selectively purify specific types of translating complexes, we conducted a one-step Flag agarose purification of our complexes prior to our AUC analyses using strains containing different Flag-tagged translational factors [11]. The Flag-tagged factors studied were Flag-PAB1, eIF4E-Flag, RPL25A-Flag (component of the 60S ribosome), and Flag-SBP1 [11, 12, 15]. Each of these purifications would purify different pools of ribosomal-associated material. The 80S free ribosome would be purified using RPL25A-Flag [11, 14, 16], whereas Flag-PAB1 purified translating complexes [11, 15, 17, 18] containing mRNA with poly(A) tails of at least 24 A’s [19], the minimal size to which PAB1 binds [20]. eIF4E-Flag and Flag-SBP1 would purify complexes containing eIF4E and SBP1, respectively, irrespective of whether the translational complexes contained a poly(A) tail. In the case of Flag-PAB1, co-expression of Flag-PAB1 with PAB1-GFP did not result in appreciable PAB1-GFP material co-immunoprecipitating with Flag-PAB1. This result implies that in our experiments only one PAB1 was present per mRNA and that bulk poly(A) tail lengths were in the range of 24 to below 48 A’s, a value in agreement with numerous studies of individual mRNA [14, 21–27]. In particular, an analysis of all PAB1-associated mRNA found that bulk poly(A) length ranged from 25 to 37 A’s [19].

**Fig 1:**
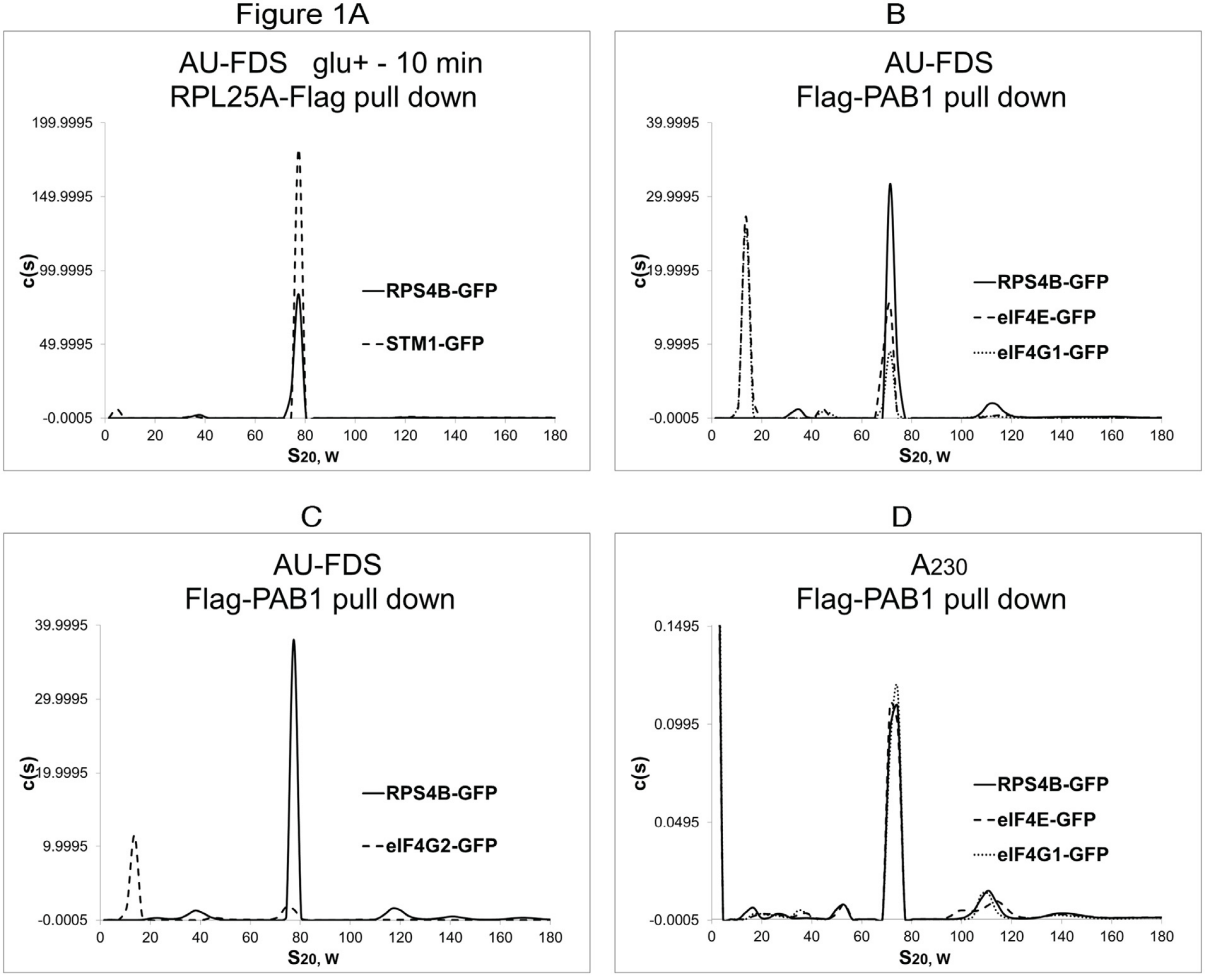
AU-FDS and AU-A_230_ analyses of extracts containing GFP fusions to translation components. For each set of data shown in Fig 1, the AUC analysis was from the same centrifuge run. Cells were grown on glucose-containing medium except as indicated. glu + - 10 min: growth was on glucose-containing medium followed by growth on medium depleted for glucose for 10 min; and glu +—+: growth was the same as glu + - 10 min except glucose was added back for 1 min. In panel A, it should be noted that the AU-A_230_ abundance for the RPS4B-GFP sample was twice that of the STM1-GFP sample. A. RPL25A-Flag pull downs were conducted on strains carrying either RPS4B-GFP or STM1-GFP; B-D. Flag-PAB1 pull downs were conducted on strains carrying the GFP fusions as indicated. Data displayed in panels B and D were done on the same day on extracts split between the two centrifuge runs.

The abundances of factors within the monosomal 77S complex were standardized to the STM1 protein that has been found to be present in a 1:1 ratio with the 80S free ribosome under glucose depleted conditions when mRNA translation has been repressed [28]. STM1 is considered to hold the 80S free ribosome together through its binding to the channel where mRNA binds; this would prevent the ribosome associating with the mRNA [28, 29]. For ease of subsequent analysis, because STM1 abundance in the pool of ribosomes would change once translation resumed, we calculated, after purifying the 80S free ribosome under glucose-depleted growth conditions using RPL25A-Flag, the ratio of STM1 to RPS4B (present in a 1:1 ratio in each ribosome of which RPS4B is a part, irrespective of growth condition) (Fig 1A). We found that the ratio of STM1 to RPS4B in the 80S free ribosome was 3.6:1 under conditions in which glucose had been depleted from the medium (Table 1). In this way, by following RPS4B abundance, in subsequent experiments, as compared to that of other translational factors, we could determine the abundance of these factors per ribosome.

**Table 1:** Relative levels of proteins in the 77S monosomal translating complex during different stages of translation.

	Flag-PAB1		eIF4E-Flag		SBP1-Flag
Factor	Initiation	Elongation	Initiation	Elongation	Elongation
**mRNA**	100	100	100	100	100
**80S ribosome**	100	100	100	100	100
**PAB1**	100	100	15 ± 1.4	12 ± 0.69	< 0.3
**eIF4E**	23 ± 1.7	16 ± 0.67	100	100	< 0.3
**eIFG1**	8.7 ± 1.0	5.6 ± 0.56	20 ± 2.4	19 ± 0.78	< 0.3
**eIF4G2**	6.2 ± 0.44	3.9 ± 0.028	15 ± 0.32	15 ± 3.4	< 0.3
**eRF1**	0.97 ± 0.069	0.97 ± 0.083	N.D.	2.8 ± 0.39	< 0.3
**SBP1**	3.3 ± 0.021	2.1 ± 0.44	N.D.	< 0.3	100
**SSD1**	1.8 ± 0.011	1.0 ± 0.051	N.D.	N.D.	N.D.
**SLF1**	3.5 ± 0.015	2.5 ± 0.17	N.D.	N.D.	N.D.
**PUB1**	N.D.	< 0.3	N.D.	N.D.	N.D.

The values represent the average (± Standard Error of the Mean, S.E.M.). N.D.- Not determined. Three to six independent replicates were analyzed except for Flag-PAB1 analyses of SSD1, SLF1, and PUB1 in which duplicates were analyzed. The number of mRNA was set at 100. The ratio of mRNA to 80S ribosomes present in the 77S complex was determined by first calculating the ratio of STM1 to RPS4B in the 80S free ribosome purified with RPL25A-Flag [11]. This value under glucose deprivation conditions was found to be 3.6 ± 0.20 (three replicas). Assuming one STM1 molecule per 80S ribosome under glucose-depleted conditions [28], we used this ratio of 3.6 for standardizing RPS4B abundance to that of each ribosome. A slightly lower ratio of STM1 to RPS4B was found under glucose growth conditions (3.0 ± 0.15) (six replicas), which is expected as the 77S monosomal translating complex migrates where the free 80S migrates and more 77S complex would be present under glucose growth conditions than under glucose-depleted conditions [11]. For RPL25A-Flag purified material we estimate, based on the abundance of PAB1, eIF4E, and eIF4G that migrates at 77S, that about 80 to 90% of the material present at 77S is free 80S ribosome, an important ratio relevant to understanding what is misleadingly termed the monosomal complex that is identified by typical sucrose gradient analysis. Elongation conditions refer to cells grown to mid-log phase on 2% glucose, which represents steady-state translational conditions in which it is assumed that the bulk of the translating ribosomes are in an elongation state across the mRNA. Initiation conditions refer to cells grown first on 2% glucose, then depleted for glucose for 10 min to repress translation, and finally subjected to re-initiation of translation with the addition of 2% glucose for 1 min.

Using Flag-PAB1, Fig 1B and 1C display representative AU-FDS analyses and the relative abundance of the closed-loop factors eIF4E, eIF4G1, and eIF4G2 found in the polyadenylated 77S monosomal translating complex relative to RPS4B. The results from AU-A_230_ analysis informed that relatively equal amounts of material were analyzed for each comparable AU-FDS run (Fig 1D, for example). Detailed analysis of fifty-one experiments indicated that preparation reproducibility between samples purified on the same day was near 1 (1.02 ± 0.028).

Table 1 gives the values under glucose growth conditions (a proxy for steady-state translational elongation) of the stoichiometry of factors known to be present in the 77S monosomal translating complex following purification of Flag-PAB1. Our data showed that for every 100 monosomal translating complexes carrying a poly(A) tail only 16% contained eIF4E and even less, about 9.5% carried eIF4G (both eIF4G1 and eIF4G2) (Table 1). Since in current models of eukaryotic translation, only one eIF4E interacts with the bridging protein eIF4G, our results demonstrate that substantial amounts of the eukaryotic initiation factors eIF4E and eIF4G are not present with polyadenylated mRNA during the elongation process for a single translating ribosome and that eIF4G is less likely to be present than eIF4E. eRF1 was found to be present in only about 1 of every 100 monosomal translating complexes. Similarly low levels of SBP1, SLF1, and SSD1 were also present in the 77S monosomal translating complex (ranging from 1 to 2.5 for every 100 mRNA), while PUB1 levels were too low to be quantitatively determined (Table 1). This low level of eIF4E/eIF4G in monosomal translating complexes is consistent with previous results that indicated that translating pools of mRNA that contain PAB1 correspondingly often do not have eIF4E/eIF4G present [19].

### eIF4E and eIF4G are preferentially present in the 77S complex at the beginning of translation

**Fig 2:**
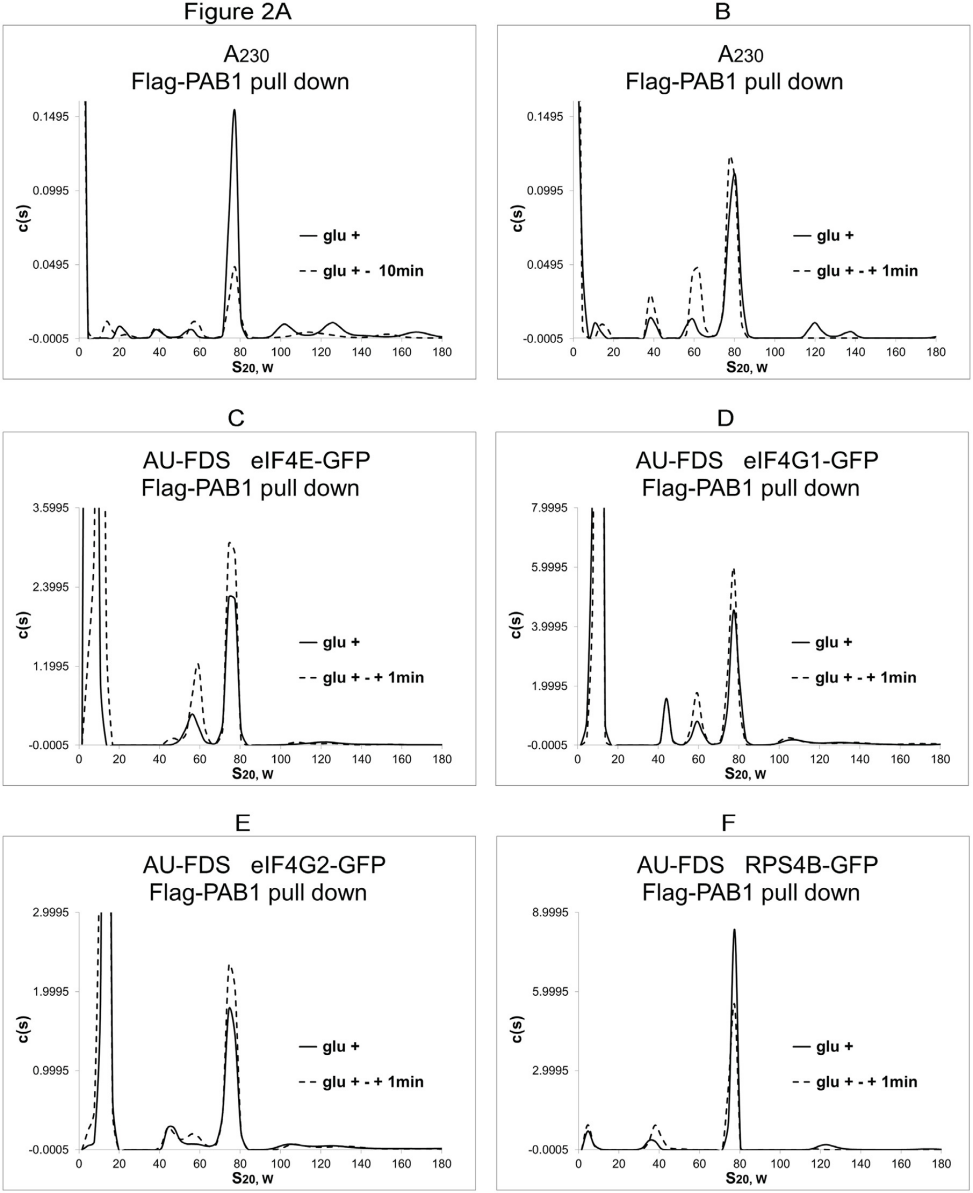
Comparison of abundance of translation factors between initiation and elongation conditions. Growth conditions for initiation conditions (glu +—+ 1 min) were obtained by adding glucose back to glucose depleted cells for 1 min, at which time cycloheximide was added and cells were harvested. Elongation conditions (glu +) were cells grown on glucose growth conditions. A-F. GFP fusions were as indicated.

In order to address the question as to how the intracellular abundance of these translation factors involved in the polyadenylated 77S monosomal translating complex changes during the initiation phase of translation, we used the approach of assaying translation initiation by adding glucose to glucose depleted cells which have arrested their translation processes [30]. Depletion of glucose significantly reduces the abundance of the 77S monosomal translating complex to 36% (S.E.M. of 1.7%, 15 determinations) of what is observed during steady-state growth, decreases polysomal abundance (glucose depleted cells contain 46% ± 4.9% of the polysomes as found under elongation conditions, 19 samples), and results in significant translational cessation [11, 30]. Adding glucose back for 1 min to arrested cells is known to increase the polysomal levels and re-initiate translation immediately [30]. We found that this treatment restored the levels of the 77S monosomal translating complex to that observed under glucose growth conditions (the abundance of the 77S monosomal translation complex upon re-initiation was found to be 94% ± 2.1%, for 37 samples, of the abundance of the 77S complex found under steady-state growth conditions) (Fig 2A and 2B). Therefore, increases in specific proteins that were observed upon re-adding glucose for 1 min would be substantially the result of new accumulation of those proteins in the reformation of new translating ribosomes. The relative abundance of polysomal material (S values from about 90S to 200S) under our initiation conditions, while increased from repressed conditions, was still only 62% (S.E.M. of ± 7.7% for 32 samples) of that found under elongation conditions. This result is expected, as a decreased number of polysomes should be present at the beginning of translation prior to reaching steady-state elongation conditions.

Our subsequent stoichiometric analysis for the polyadenylated 77S monosomal translating complex under these initiation conditions indicated that the absolute abundances of eIF4E, eIF4G1, and eIF4G2 increased by about 1.6-fold in the 77S complex after re-addition of glucose for 1min to the glucose-starved cells as compared to steady-state elongation conditions (Fig 2C to 2E as compared to RPS4B, Fig 2F; summarized in Table 1). These data are in agreement with the translational model in which eIF4E and eIF4G should be more present for a ribosome beginning translation. Importantly, for the monosomal translating complex, even at initiation, a significant pool of PAB1-containing translating mRNA lacked both eIF4E and eIF4G. We also observed that eRF1 displayed no increased abundance in the 77S monosomal translating complex at the very beginning of translation (Table 1), which is consistent with the role or eRF1 in termination. Surprisingly, however, the known translation repressor SBP1 was observed to be 1.6-fold more abundant in the 77S monosomal translation complex during initiation than elongation (Table 1).

**Fig 3:**
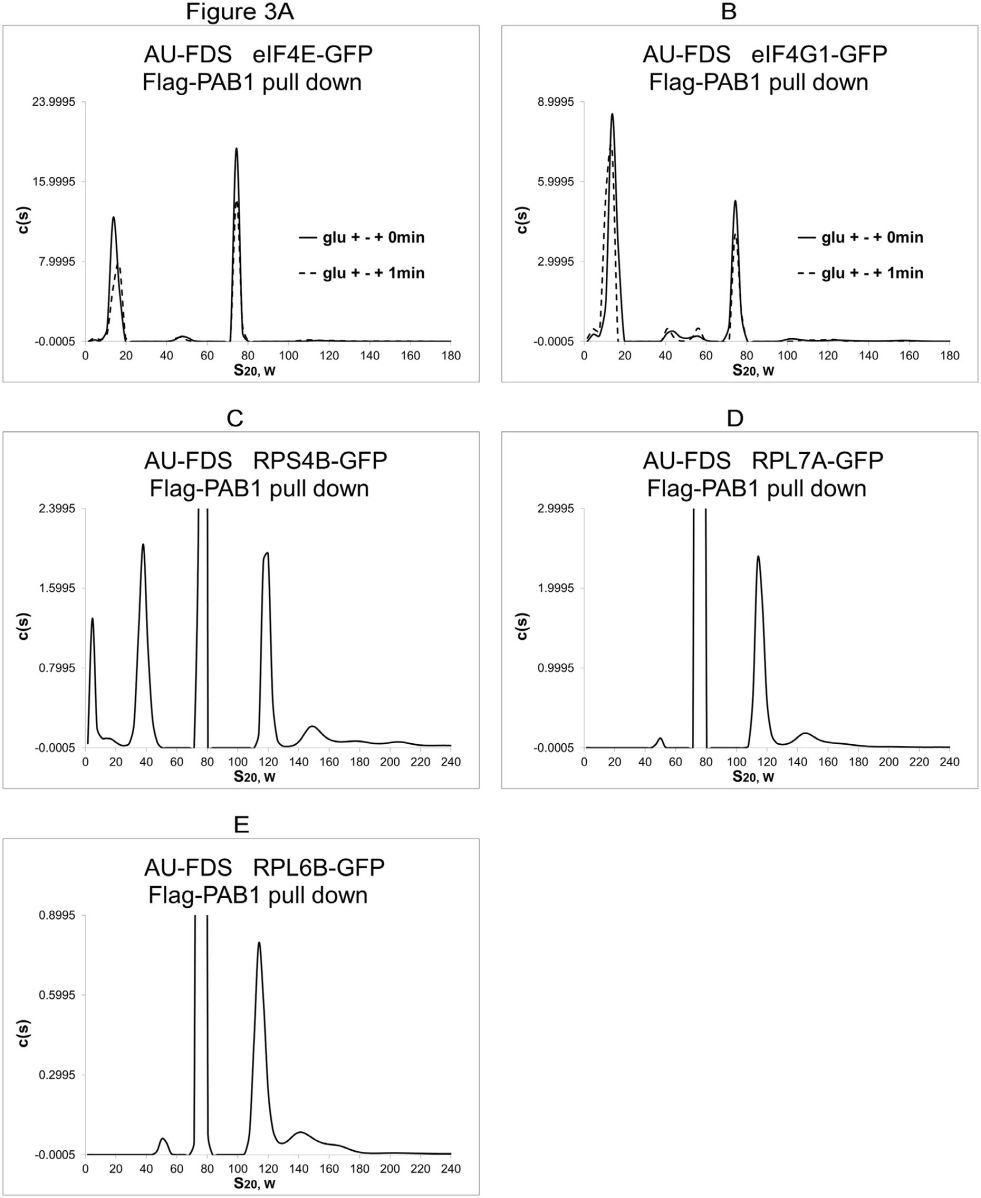
AU-FDS analysis of two initiation conditions and polysomes. A and B. Comparison of two different initiation conditions. C-E. Expanded c(s) values to identify ribosomal-GFP protein migrations in polysomal material (greater than 90S).

To further verify that our conditions were indeed assaying initiation, we backed up the time of re-adding glucose to glucose starved cells to occur at 0 min and at the same time we added cycloheximide to freeze translating ribosomes. Because of the time delay in action of cycloheximide, we hypothesized we would be catching cellular initiation events just prior to our 1 min time point described above, which would be closer to observing newly formed translation complexes. The absolute abundances of eIF4E and eIF4G1 were found to increase in the polyadenylated 77S monosomal translating complex at 0 min after adding glucose as compared to 1 min: a 42% increase (S.E.M. of ± 1.6%) and 29% (± 0.66%), respectively. These results confirm our hypothesis that re-initiation events were being identified by these procedures (Fig 3A and 3B). However, even under these earlier initiation conditions, the abundance of eIF4G was less than that of eIF4E, implying decreased levels of eIF4G relative to eIF4E in the monosomal translating complex even at the commencement of translation.

### During elongation eIF4E and eIF4G are preferentially associated in polysomes

We subsequently determined the absolute abundance of closed-loop factors in the translating polyadenylated polysomes (material that migrates from 90S to 200S), which consists primarily of disomes (110 to 130S), some trisomes (130 to 160S), and much more minor contributions from tetrasomes (160 to 200S), using the same methodology as described above with Flag-PAB1 as our handle (Fig 3C to 3E). We estimated the average number of ribosomes per mRNA for the 90S to 200S region for Flag-PAB1 pull downs by calculating the relative abundances of different ribosomal subunits (RPS4B, RPL7A, and RPL6B) in disomal, trisomal, and tetrasomal peaks in this region. We found that the mean number of ribosomes per mRNA for 38 analyses was 2.4 ribosomes (S.E.M. of ± 0.060). As previously described [11], for reasons that are unclear AUC analysis of polysomes does not adequately identify larger polysomal complexes than pentasomes and under detects the actual levels of polysomes present as compared to the canonical sucrose gradient centrifugation analysis. However, other types of macromolecules of S values greater than 90S are readily identified following AUC analysis. Formaldehyde cross-linking of polysomes in vivo prior to cell lysis did not assuage this issue [12], indicating that the somewhat ineffective visualization of polysomes by AUC analysis as compared to sucrose gradient centrifugation analysis was not due to specific breakdown, conversion, or degradation of polysomal complexes. This lack of visualization of polysomes is not due to the inability of Flag agarose beads to purify polysomal complexes, as we observe the same low level of polysomes when crude extracts are analyzed by AUC analysis [11]. The most likely cause for this effect is the different centrifugation methodologies used between AUC analysis and sucrose gradient analysis. In the latter case polysomes are condensed into increasing greater concentrations of sucrose, possibly creating artificial associations.

**Fig 4:**
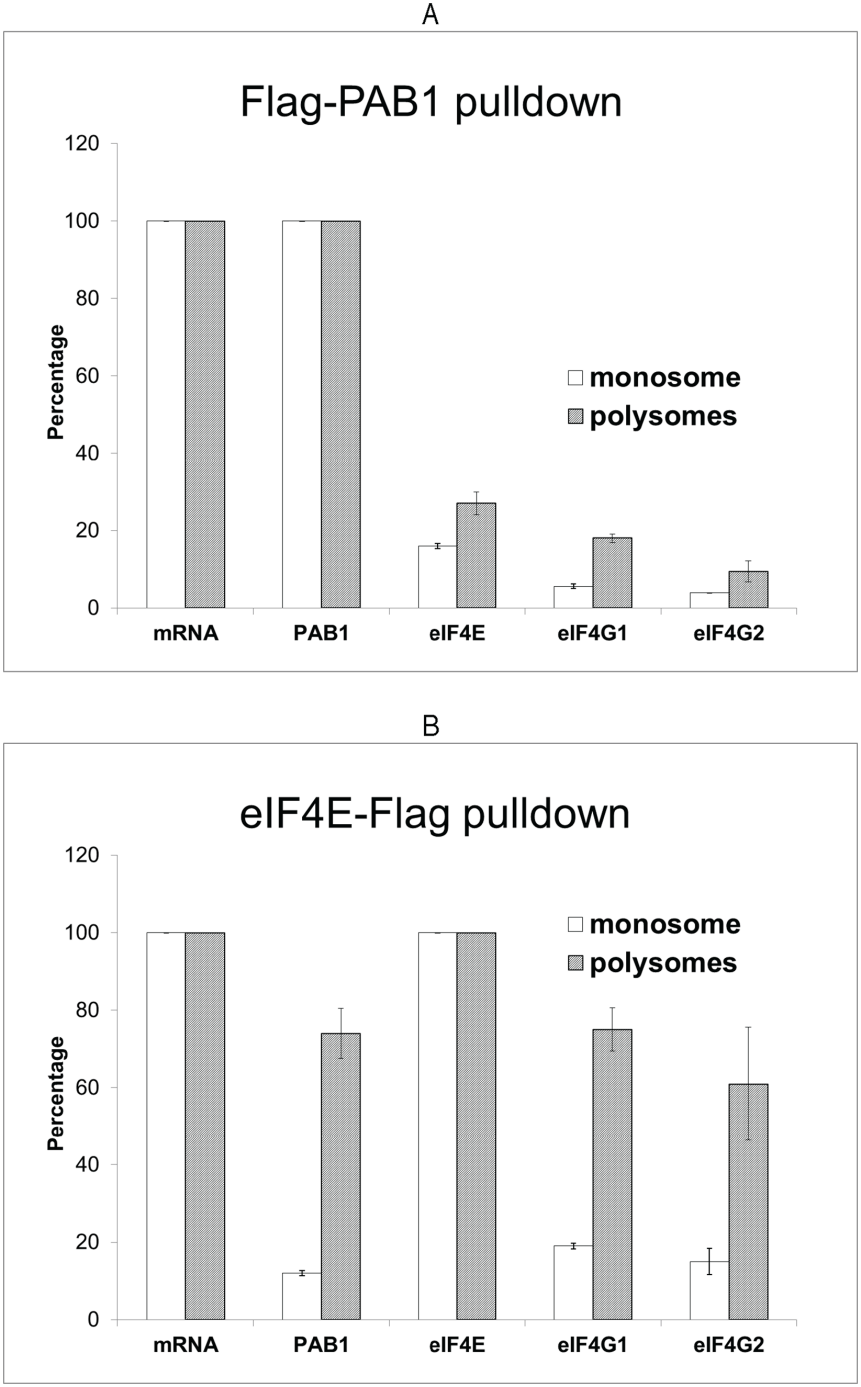
Percentage of translation factors in polysomal versus monosomal complexes. Elongation conditions were as described in Table 1. The mRNA abundance was set at 100%. For a given Flag pull down, the translation factor tagged with Flag was also set at 100%. A. Flag-PAB1 pull downs; B. eIF4E-Flag pull downs.

During steady-state elongation, 27% of these ribosomes in polysomes contained eIF4E (Table 2), a very significant increase over that observed for the monosomal translating complex (Table 1 and Fig 4A). Most noteworthy, in these polysomes the total eIF4G abundance was comparable to that of eIF4E, 27%, implying that in polyadenylated polysomes 100% of the eIF4E-containing complexes were associated with eIF4G. eRF1 levels, in contrast, did not increase in polysomes compared to monosomes and, in fact, were found to be extremely low (Table 2). These results suggest that for mRNA with poly(A) tails eIF4G is more stabilized in the closed-loop structure in polysomes than it is in monosomal translating complexes.

**Table 2:** Relative levels of proteins in the polysomal translating complexes during different stages of translation.

Protein/mRNA	Flag-PAB1 Initiation	Flag-PAB1 Elongation	eIF4E-Flag Initiation	eIF4E-Flag Elongation
**mRNA**	100	100	100	100
**80S ribosomes**	240	240	210	310
**PAB1**	100	100	59 ± 11	74 ± 6.5
**eIF4E**	38 ± 3.0	27 ± 3.0	100	100
**eIF4G1**	26 ± 4.8	18 ± 1.1	79 ± 25	75 ± 5.6
**eIF4G2**	13 ± 0.87	9.4 ± 1.2	56 ± 14	61 ± 14.6
**eRF1**	< 0.3	< 0.3	N.D.	< 0.3
**SBP1**	11 ± 1.7	8.3 ± 1.9	N.D.	< 0.3

The values for the various translation factors in the polysomal material were determined as described in the Legend for Table 1. Initiation and elongation conditions were as described in Table 1 and the text. For the AU-FDS analysis, individual polysomal complexes were not identified and instead the abundance of all material ranging from 90S to 200S was considered “polysomal.” To determine the actual average distribution of ribosomes in this range, we followed RPS4B-, RPL6B-, and RPL7A-GFP to identify discrete disomal, trisomal, and tetrasomal material. The relative abundance of each of these fluorescent peaks was used to calculate the number of ribosomes per mRNA present in the 90S to 200S region.

A similar stoichiometric analysis of closed-loop components in polyadenylated polysomes under initiation conditions was also conducted. As was observed for the monosomal translating complexes, an increased abundance of closed-loop factors was present in polyadenylated polysomes under initiation conditions as compared to elongation conditions (about 40% more) (Table 2). Again, we observed that for polyadenylated polysomes under initiation conditions the total levels of eIF4G were essentially equivalent to that of eIF4E, implying that eIF4G presence in the closed-loop structure is stabilized by polysomal structure. While SBP1 levels in Flag-PAB1 purified polysomes also appeared to increase under initiation conditions as compared to elongation conditions, this increase did not appear to be significant. These results also indicate that PAB1-associated translating mRNA, even if they are polysomal, often lack eIF4E and eIF4G.

### eIF4E-containing translation complexes preferentially contain eIF4G

**Fig 5:**
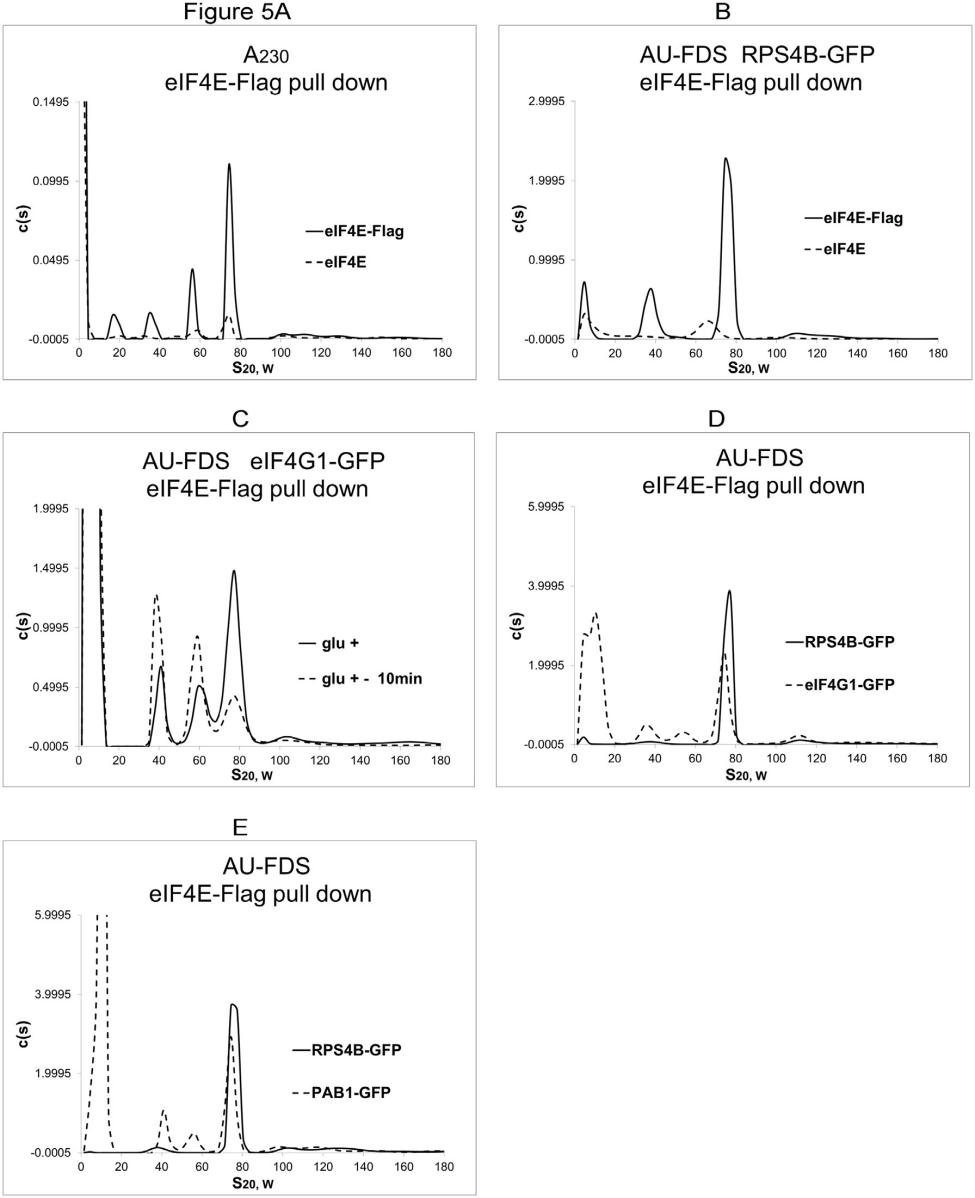
Analysis of eIF4E-Flag purified translation complexes. Growth conditions and analysis were conducted as described in Fig 1. A and B. Strains were either transformed with eIF4E-Flag plasmid (eIF4E-Flag) or with no eIF4E plasmid (eIF4E).

We subsequently used eIF4E-Flag to immunoprecipitate monosomal and polysomal complexes to address the composition of closed-loop components when eIF4E was present even if the mRNA were substantially deadenylated. A 77S complex was detected using eIF4E-Flag pull downs followed by AU-A_230_ analysis (Fig 5A) or AU-FDS analysis (Fig 5B for RPS4B-GFP) when compared to control pull-downs with strains containing only eIF4E. This putative 77S complex was shown to become reduced in abundance following growth on glucose depleted media, as expected for a 77S translating monosomal complex (see, for example, Fig 5C). Table 1 summarizes the stoichiometric ratios under glucose steady-state conditions for the abundance of factors found in the 77S monosomal translating complex as purified with eIF4E-Flag (representative data in Fig 5D and 5E).

Several observations can be made from this data. First, only 12% of the eIF4E-containing complexes contained PAB1, a result suggesting that the bulk of the eIF4E-mRNA translating monosomal complexes have mRNA with poly(A) tails shorter than 24 A’s. This observation is consistent with previous results that demonstrated that eIF4E- and eIF4G-containing bulk mRNA have shorter poly(A) tails than PAB1-associated mRNA [19]. While it is possible that PAB1 protein is dissociating from the poly(A) tail upon cell lysis, this seems very unlikely. For instance, PAB1 associates very strongly with the poly(A) tail with a *K*_*d*_ of 11 nM [18], even 2 M KCl salt washes dissociate only about 50% of PAB1 bound to poly(A) sepharose [17], and in vivo formaldehyde cross-linking of translating complexes did not result in more Flag-PAB1 being detected in translating complexes as compared to cells not cross-linked prior to cell lysis [12]. Second, for the 77S monosomal translating complex, 33% of the eIF4E complexes also have eIF4G bound to them. These first two observations suggest a closed-loop structure on the mRNA that can be formed between eIF4E and eIF4G even in the absence of PAB1. This conclusion is consistent with previous studies that indicate that eIF4G can bind mRNA, that these interactions contribute significantly to translation [8–10], and that eIF4E/eIF4G mRNA complexes often lack PAB1 [19]. Moreover, these results are in agreement with the recent observation that more mRNA actually form a closed-loop structure containing solely eIF4E and eIF4G than do they form the canonical eF4E-eIF4G-PAB1-mRNA closed-loop structure [31]. Since eIF4G appears to be as equally represented in polysomal material as is PAB1 (Fig 5) [11, 32], this eIF4E-eIF4G-mRNA closed-loop structure would be important to the translation of many mRNA. Third, no significant levels of SBP1 were found associated with eIF4E, suggesting that the two proteins are present in mutually exclusive pools of 77S monosomal translating complexes. Fourth, using eIF4E-Flag expressed in a strain carrying eIF4E-GFP, we were unable to purify any eIF4E-GFP material migrating at 77S. This result implies that there is only one eIF4E molecule per mRNA in agreement with current theories.

Similar analyses for the abundance of factors in eIF4E-containing complexes were conducted under the translation initiation conditions described above in which glucose was added back for 1 min to glucose depleted cells. In this case, we observed a 25% increase in the number of eIF4E-containing 77S monosomal translating complexes that carried PAB1, but no significant increase in the number of complexes containing eIF4G (Table 1). The observation that the eIF4G to eIF4E ratio remains constant as translating complexes move from initiation to elongation is consistent with the observations described for Flag-PAB1 pull downs. In that case, the ratio of eIF4G to eIF4E also remained constant when initiation conditions were compared to elongation conditions, as both factors dissociated from the monosomal translating complex to the same relative extent (60% reduction) during the translation process (Table 1). Yet, even at initiation the majority of the 77S monosomal translating complexes we observe with eIF4E-Flag are lacking PAB1 and eIF4G. These observations are consistent with the model that eIF4E-containing monosomal translating complexes contain poly(A) tails shorter than 24 A’s and are moving towards translational cessation.

When the stoichiometry of factors in polysomes was analyzed following eIF4E-Flag pull downs, much different results were obtained than with the 77S monosomal translating complex (Table 1; Fig 4B). For polysomes after eIF4E-Flag pull downs, 74% of the ribosomes carried PAB1, a number much greater than that found for monosomal translating complexes purified with eIF4E-Flag (12%), indicating that most mRNA in such complexes contain poly(A) tail lengths of at least 24 A’s. Moreover, 75% of eIF4E-Flag-containing polysomes carried eIF4G1 and another 61% had eIF4G2, again greater than that found in monosomal translating complexes (33%). This number of eIF4G molecules in polysomes is greater than the presumed number of eIF4E present, suggestive of more than one eIF4G molecule per polysomal complex. While the Discussion elaborates on other possible causes for this result (including minor inaccuracies in our determination of absolute abundances), the fact that in Flag-PAB1 pull downs the ratio of eIF4E to eIF4G for polysomes is actually 1:1 under both initiation and elongation conditions suggests there is no error in calculating absolute abundances. This implies that the higher ratio of eIF4G to eIF4E found in polysomes identified with eIF4E-Flag pull downs represents the presence of more than one eIF4G molecule per mRNA undergoing translation. Even with the more conservative interpretation of these results, the high number of eIF4G molecules present in eIF4E-containing polysomes supports at least a 1:1 correspondence between eIF4E and eIF4G in polysomes. We also found that in the eIF4E-Flag pull downs the number of ribosomes per mRNA present in our pool of polysomes from 90S to 200S was greater (3.1 ± 0.27 ribosomes per mRNA, 15 samples) than for those from Flag-PAB1 pull downs (2.4 ribosomes per mRNA). This result implies that polysomal structure is more correlated with eIF4E/eIF4G presence than it is with poly(A) tails. It should also be noted that as shown for the monosomal translating complexes there exists a significant pool of polysomes (about 25 to 40%) that appear to be substantially deadenylated and yet still bound by eIF4E and eIF4G. This observation again supports the wide prevalence of the alternative eIF4E-eIF4G-mRNA closed-loop structure that has been observed in vivo [31].

For polysomes purified with eIF4E-Flag under initiation conditions, the relative abundance of PAB1 and eIF4G remained about the same as observed under steady state growth conditions, consistent with particular stabilization of eIF4E-eIF4G interactions in polysomal structures (Table 2). Again, eIF4G levels were greater than the abundance of eIF4E, suggesting that in polysomes more than one eIF4G molecule per mRNA can be present. The observation that the ribosomal density per mRNA was less at initiation of translation (2.1) compared to elongation conditions (3.1) is consistent with our initiation conditions identifying an early stage in translation prior to full ribosomal occupancy of mRNA.

### Translational complexes containing the SBP1 repressor lack poly(A) tails and eIF4E/eIF4G

**Fig 6:**
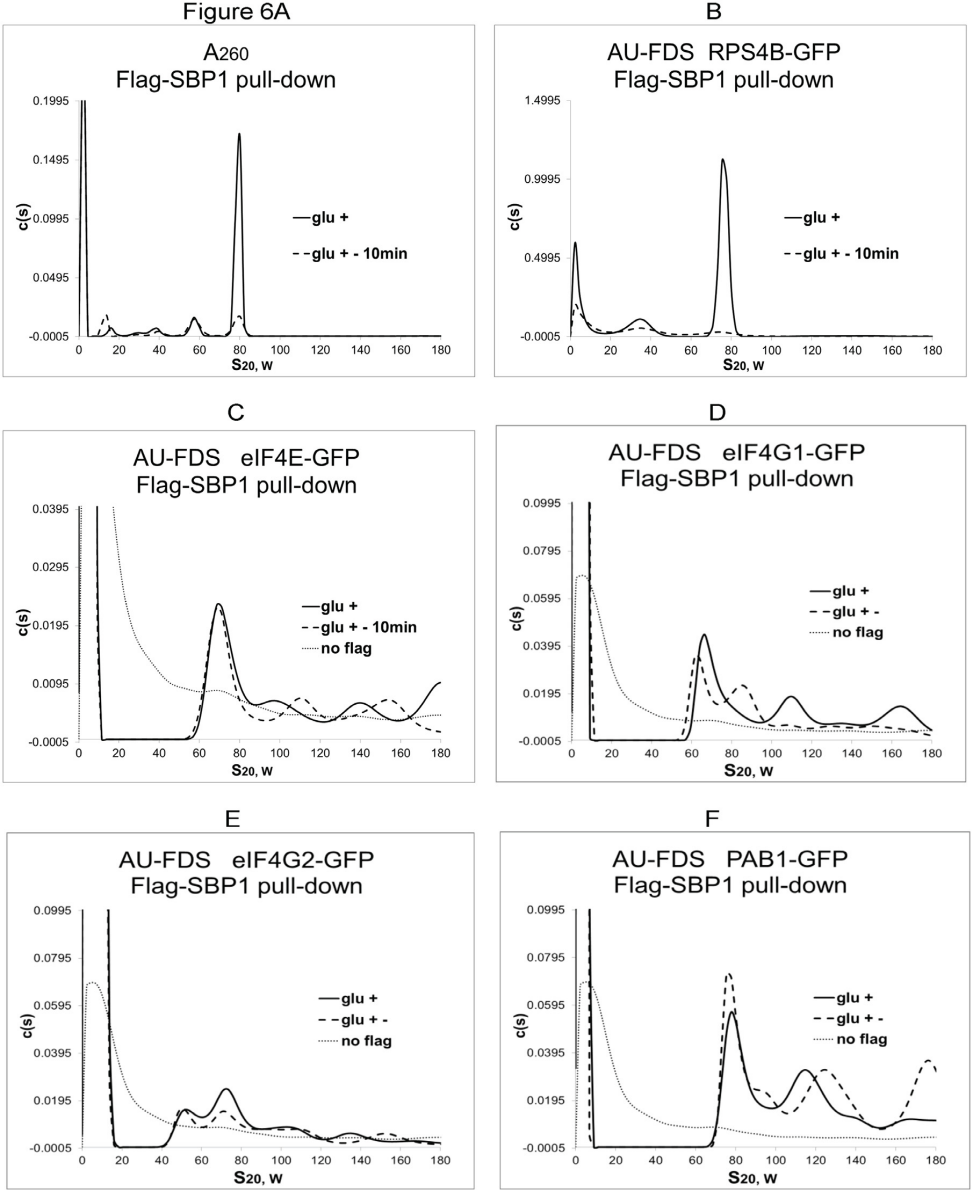
Analysis of Flag-SBP1 purified translation complexes. A. AU-A_260_ analysis was conducted instead of AU-A_230_ analysis for better detection of complexes with this particular Flag-tagged protein. A-F. Growth conditions were as described in Fig 1. C. No flag refers to a strain lacking Flag-SBP1 plasmid. The differences between the no flag control and the Flag-SBP1 pull downs in panel C-F were not considered significant for eIF4E-GFP and eIF4G2-GFP.

Because SBP1 was not found to be present in eIF4E-containing complexes, we addressed what types of translational complexes Flag-SBP1 could pull down. SBP1 could bring down a 77S complex (76.4S ± 2.68S, average of five determinations) that became reduced in abundance following translational cessation caused by glucose depletion (Fig 6A and 6B), indicating that the 77S complex is the monosomal translating complex [11]. The relative abundances of translation initiation factors present in this complex were determined and indicated that SBP1-containing complexes displayed very low levels of PAB1 and eIF4E/eIF4G1/eIF4G2 in a complex migrating around 80S (Fig 6C–6F; Table 1). In that the abundances of these low abundant PAB1/eIF4E/eIF4G complexes migrating at 80S were unresponsive to glucose depletion, they do not represent the 77S monosomal translating complexes. These results imply that SBP1 is bound to mRNA complexes that are substantially deadenylated and which also lack eIF4E/eIF4G. It was also observed that Flag-SBP1 failed to bring down significant levels of polysomal complexes (Fig 6A and 6B), suggesting a role for SBP1 in a late stage of translational shut off.

## Discussion

We have used AU-FDS analysis of GFP-tagged translation factors to quantitate the abundance of translational factors in two types of translational complexes: the 77S monosomal translating complex and polysomes. To increase the robustness of our analysis, three different handles were used to purify these complexes: PAB1, eIF4E, and SBP1. Each of these handles offered gateways to identifying different types of translational complexes. The identification of complexes at two different stages of translation were also monitored, initiation and steady-state elongation.

**Fig 7:**
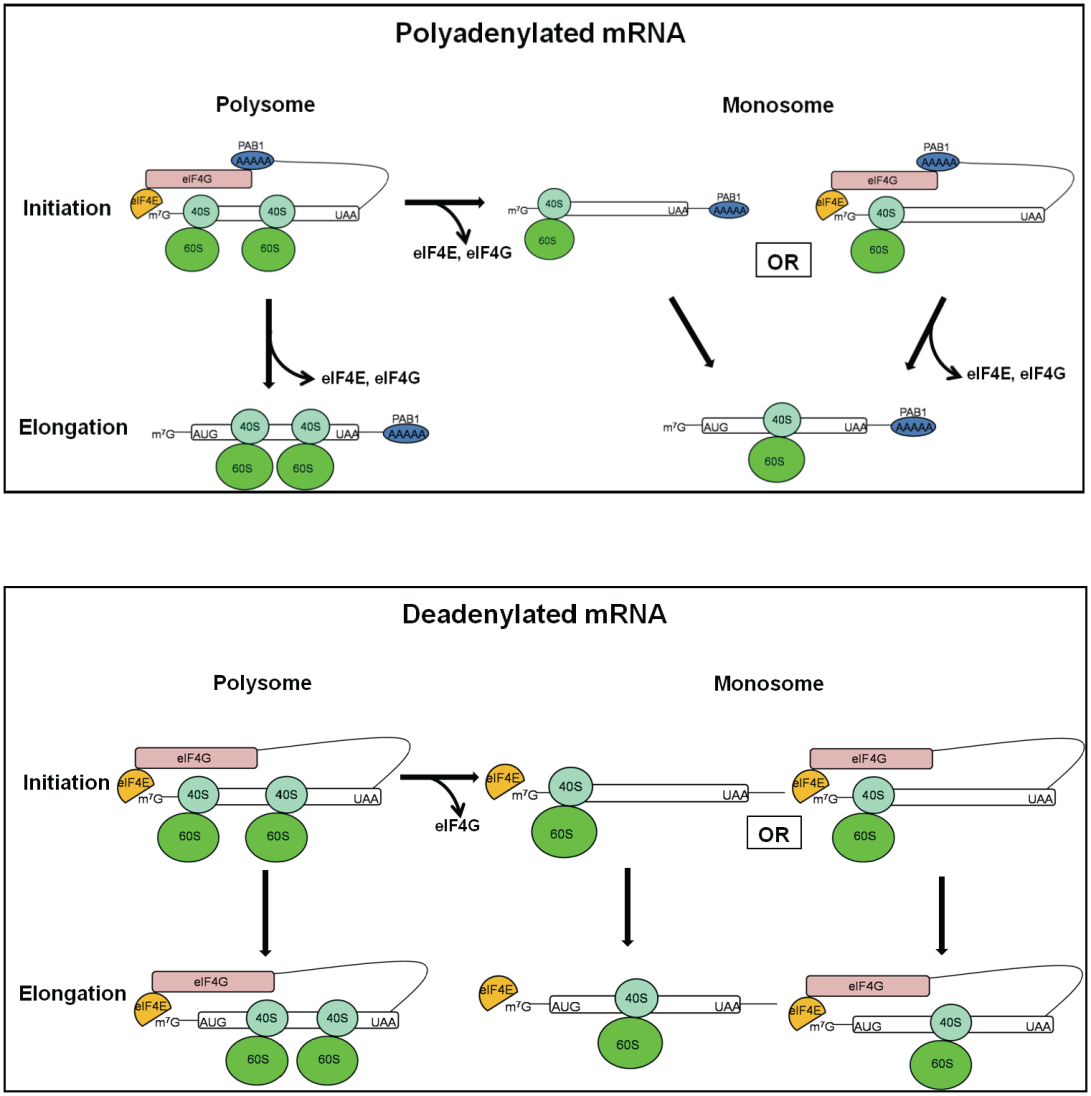
Summary of dynamic changes in closed-loop factors in translating ribosomes dependent on polysomal, polyadenylation, initiation, and elongation states. Only the translating ribosomes at initiation containing closed-loop structures are summarized. For simplicity, the dynamic changes of closed-loop factors in translating ribosomes containing other combinations of closed-loop factors are not represented, and these changes in stoichiometry are explained more completely in the text. Also, based on our results only one PAB1 is represented as binding to each poly(A) tail.

A number of major conclusions can be reached from these analyses. First, for polyadenylated mRNA (poly(A) lengths greater than 23 A’s), both eIF4E and eIF4G dissociated from translating monosomes and polysomes during the movement from initiation to elongation (summarized in Fig 7). For example, 23% of the polyadenylated translating monosomal complexes at initiation contained eIF4E whereas only 16% of the elongating monosomes contained eIF4E. Similarly, for polysomal complexes, 38% of polyadenylated complexes contained eIF4E at initiation compared to 27% during elongation. eIF4G dissociation patterns were similar to that of eIF4E, implying simultaneous dissociation of these two factors As these closed-loop factors are important to the translation process, their loss, as ribosomes transit the mRNA, implicate this transition as a key regulatory target.

Second, in the transition from the polysomal state to the monosomal state two observations were made. Significant levels of eIF4E/eIF4G dissociated from the polyadenylated mRNA and for either polyadenylated or deadenylated mRNA (the latter containing eIF4E), eIF4G particularly dissociated more readily than did eIF4E (Fig 4A and 4B) (see also Fig 7). For example, for polysomes containing PAB1 during elongation, 27% had eIF4E/eIF4G whereas only 16% of translating monosomes had eIF4E and even less had eIF4G. The same pattern of results inhered under initiation conditions. Moreover, relative to eIF4E, eIF4G preferentially dissociated during the polysome to monosome transition: the eIF4E/eIF4G ratio was near 1:1 for polysomes but was about 1.6:1 for polyadenylated monosomes and 3:1 for eIF4E-containing translating monosomes. Even under initiation conditions, monosomes were more depleted for eIF4G than they were for eIF4E. Also, monosomal translating complexes containing eIF4E had six-fold less PAB1 associated with them (and hence shorter poly(A) tails) than the corresponding polysomal complexes. The combination of shorter poly(A) tails and the loss of eIF4E with even greater losses of eIF4G indicate that translating monosomes would be significantly impaired for translation as compared to polysomes, suggestive that translating monosomes are nearing the end of translation. A recent conclusion that monosomal translating complexes are slowed in the initiation of translation relative to polysomal complexes [33] is consistent with our observation of the particular depletion of eIF4E, even greater losses of eIF4G, and increased abundance of deadenylated mRNA in monosomal complexes, all consequences that would be expected to slow initiation processes. Moreover, since changes in poly(A) tail length and closed-loop factor association with the mRNA critically regulate mRNA decay rates [21, 23–27], alteration in these factors, as observed for monosomal translating complexes, would provide a mechanistic explanation for the recent observation that mRNA associated with monosomal translating complexes are particularly subject to enhanced rates of mRNA degradation [33].

Correspondingly, the observation that the eIF4E to eIF4G ratio for polysomes was at least 1:1 for either PAB1- or eIF4E-containing complexes implicates polysomal structure as particularly stabilizing to the closed-loop structure involving eIF4E, eIF4G, and PAB1 or to that of just eIF4E and eIF4G. It is unclear, however, if this stabilization effect is due to a particular alignment of multiple ribosomes with the mRNA and the closed-loop factors that translating monosomal complexes cannot provide or to these closed-loop complexes promoting polysomal structure, that is, by enhancing ribosome re-initiation on mRNA.

Third, the observation that eIF4G preferentially dissociated during the polysome to monosome transition suggests further that eIF4G association with translating complexes is a preferred target by translational repressors. This possibility is consistent with the identification of several important translational regulators that bind to and possibly release eIF4G from translating complexes [34]. SBP1 has been suggested to be one of these translational repressors [34]. The role of eIF4E-binding proteins that compete with eIF4G interaction with eIF4E may also play essential roles in aiding eIF4G-preferential dissociation from translation complexes [1].

Fourth, for both translating monosomes and polysomes, a significant number of complexes lack PAB1 but still carry eIF4E/eIF4G. In that eIF4G can make RNA contacts important to translation independent of its binding to PAB1 associated with the poly(A) tail [8–10, 35], these eIF4E/eIF4G complexes appear to be forming a closed-loop structure that is highly competent for translation even in the absence of PAB1. Such closed-loop structures would add an additional level of redundancy to translational processes [1], especially for those mRNA whose poly(A) tails have been shortened below the limit for competent PAB1 binding. Deadenylated mRNA are found associated with translating polysomes [14] (consistent with the importance of this alternative closed-loop structure to translation), and many mRNA are associated with eIF4E/eIF4G but not with PAB1 [32].

Most importantly, as presented in Tables 1 and 2 and summarized in Fig 7, polysomal and monosomal translating complexes that are essentially deadenylated (and which contain eIF4E and eIF4G in presumably a closed-loop structure) are particularly insensitive to eIF4G loss as ribosomes transit the mRNA from initiation to elongation in contrast to what is found for polyadenylated mRNA. This implies that the eIF4E-eIF4G-mRNA closed-loop structure provides an increased stability not observed with the canonical eIF4E-eIF4G-PAB1-mRNA closed-loop structure. The implications from this may be very significant. First, it has been shown that a sizeable portion of translating mRNA are sensitive to eIF4G depletion even for mRNA containing shortened poly(A) tails that presumably lack PAB1 [36]. These observations support the importance of an eIF4E-eIF4G-mRNA closed-loop structure to translation of specific mRNAs that has been verified by in vivo analysis of a number of mRNA [31]. Second, our data imply that significant and stable translation of deadenylated mRNA is occurring in the cell, as shown previously [14]. Specific mRNA species, including those of the histone mRNAs that are lacking poly(A) tails (which may be using an eIF4E-eIF4G-eIF3-SLIP1-mRNA closed-loop structure) [37, 38], may be, in addition, particularly using the eIF4E-eIF4G-mRNA closed-loop structure, again implying specific regulation of such structures. Third, in that certain viral infections, such as those mediated by HIV, coxsackieviruses, enteroviruses, and poliovirus, particularly target PAB1 for degradation [39–41], the prevalence of an alternative closed-loop structure would be a critical part of the translation process in both normal and disease states in terms of regulation and the important resistance to and progression of these environmental impacts.

Importantly, our data indicate that translational complexes are not monolithic. For example, monosomal poly(A)-containing complexes substantially lacked eIF4E/eIF4G while those that contained eIF4E predominantly lacked poly(A) tails. Polysomal poly(A)-containing complexes also had a majority of complexes lacking eIF4E/eIF4G, whereas those containing eIF4E were essentially of a closed-loop structure, containing eIF4G alone or with PAB1. These data agree with other observations that the mRNA found in PAB1-purified material can be substantially distinct from those found in either eIF4E- or eIF4G-purified material [19]. Moreover, those translation complexes that carried SBP1 were both predominantly deadenylated (poly(A) lengths shorter than 24 A’s) and lacking in eIF4E/eIF4G.

In that SBP1-containing monosomes appear to be moving towards translational repression, as they lack key closed-loop factors that augment translation, SBP1 might be playing a direct role in this repression process. One role of SBP1 in translational repression has been suggested to occur via SPB1 binding to eIF4G and affecting its translational abilities [34]. Whether SBP1 passively associates with mRNA lacking eIF4E/eIF4G or actively removes such components is not clear from our results.

The analysis of SBP1 further hints at additional different pools of translation complexes. While our results using Flag-SBP1 purified material suggest that SBP1 binding to the 77S monosomal translation complex preferentially occurs on substantially deadenylated mRNA, we also observed using Flag-PAB1-purified material that about 2.1% of the polyadenylated monosomal translation complexes and 8.6% of the polyadenylated polysomes contain SBP1. It is unlikely that these pools of translational complexes contain eIF4E/eIF4G because eIF4E-Flag pull downs were deficient in SBP1 (Tables 1 and 2). Therefore, while the vast majority of translational complexes with SBP1 contain substantially deadenylated poly(A) tails, a small percentage of mRNA still have SBP1 present in both monosomal translation complexes and polysomes that have longer poly(A) tails. Also, the abundances of both of these pools of SBP1-containing polyadenylated translation complexes were increased during initiation relative to elongation. This suggests that SBP1 may be playing an additional role in initiation processes that has not previously been detected. Whether this minority of mRNA that have both SBP1 and poly(A) tails represent a specific class of mRNA species or are found evenly distributed across all cellular mRNA remains to be determined.

Our results also imply that more than one eIF4G is bound to each translating polysome in that we observe actually more eIF4G than eIF4E in eIF4E-containing polysomal complexes. Previous studies have suggested that multiple eIF4G molecules may be present on each mRNA [42]. On the other hand, the current accepted model of the closed-loop structure is a 1:1 ratio of eIF4E to eIF4G, although there is no conclusive evidence to firmly establish this ratio. In that eIF4G can make contacts to mRNA separate from that of contacting PAB1 [8–10, 35], it remains possible that polysomes may actually carry more than one eIF4G. This might be especially critical for those polysomes that lack PAB1 in which multiple eIF4G would provide extended mRNA contacts and hence closed-loop structural advantages to translation. An alternative possibility is that we may have mis-estimated the absolute abundance of STM1 relative to RPS4B. If this value were actually only 30% higher than we calculated, then we would have obtained a nearly 1:1 ratio of eIF4G to eIF4E for polysomes identified with eIF4E-Flag. It is also possible that the replacement at the chromosomal location of either eIF4G1 or eIF4G2 by its corresponding GFP fusion could have altered the expression levels of the eIF4G1 and/or eIF4G2 protein causing our ratio of eIF4G to eIF4E to be greater than expected. Considering these alternatives, we favor the presence of more than one eIF4G molecule per translating polysome that also carries eIF4E because the ratio of eIF4G to eIF4E was exactly 1:1 for PAB1-containing polysomes under both initiation and elongation conditions (Table 2). For these polysomes involving polyadenylated mRNA there may not be a selective advantage for having more than one eIF4G present per complex, as eIF4G would be closing the closed-loop structure by contacting PAB1. In contrast, in that about 25% to 40% of eIF4E-containing polysomes lack PAB1, it may be that for these polysomes multiple eIF4G per polysome may be required for full stabilization of the closed-loop structure.

Although there may be some possible limitations (as described above) that inhere to AU-FDS in providing an absolute accurate quantitation of components within translating complexes, the reliability of the relative ratios of factors remains as well as do the conclusions derived from the changes of these ratios during translation. To clarify the changes we observed, specific mRNA will need to be actively followed during translation. For example, poly(A)-containing mRNA have previously been shown to be enriched in those mRNA that are highly expressed in cells, including those in carbohydrate metabolism [19]. Also, monosomal translating complexes have recently been shown to be enriched in mRNA that are encoding key cellular regulatory components, such as protein kinases, protein phosphatases, and transcription factors [33]. These observations imply that there are multiple factors that can influence translational progress and the association with the mRNA of key translational proteins. In that AU-FDS has allowed the detection of the 77S monosomal translating complexes not previously studied by conventional means, other novel translating complexes may be similarly detected by AU-FDS dependent on which handles are used to purify these complexes, thereby enriching the analysis of the actual translational complexes present in vivo.

## Materials and Methods

### Yeast strains and growth conditions

Yeast strains carrying GFP fusions to particular translational factors have been previously described [11, 43]. All strains except for that containing eIF4G2-GFP were isogenic with the genotype *Matα ura3 his3 leu2 met15*, with the GFP fusion marked with the *HIS3* gene [43]. The strain carrying eIF4G2-GFP was RP2384, *Mata leu2-3*,*112*, *trp1 ura3-52 his4-539 cup1*::*LEU2/PGK1pG/MFA2pG TIF4632-GFP*::*G418*, and was provided by Roy Parker. Previous studies have shown no difference in AU analysis for strains isogenic to RP2384 and to the other strains used [11]. All strains were transformed with one of the following plasmids as indicated in the text: YC776 (*Flag-PAB1 URA3*), JC288 (*RPL25A-Flag URA3*), WX03 (*Flag-SBP1 URA3*), or YC801 (*eIF4E-Flag URA3*). Cells were grown at 30°C to mid-log phase in synthetic complete medium with appropriate amino acids as described before [18]. Generally, 200 mL of cells were used for AU-FDS analyses. Cell lysis and Flag pull downs have been described [11]. Briefly, yeast cells were lysed in ice-cold Tris buffer (pH 7.5, 0.05 M Tris-base, 0.15 M KCl, 2 mM MgCl_2_, 10% glycerol, 1 mM PMSF, and a 1:500 dilution of Protease Inhibitor Cocktail (Sigma-Aldrich P8215)). Generally, 1 mL of a 15 to 25 mg/mL crude extract was incubated with anti-Flag affinity beads (Sigma-Aldrich) by gentle shaking for two hours at 4°C. After five washes with 1 mL Tris buffer, the purified samples (500 μL) were eluted twice with 200 μg/ml of Flag peptide (N-DYKDDDDK-C, Sigma) in Tris buffer (without the Protease Inhibitor Cocktail) at 4°C for 30 to 40 min. The protein concentration that was analyzed by AUC was in the 0.1 to 0.3 mg/mL range. Control experiments conducted with strains lacking a Flag-tagged protein resulted in Flag-purified protein concentrations in the 0.02 mg/mL range following Flag peptide elution.

For glucose depletion, cell pellets from the undepleted medium were washed and then resuspended in fresh medium lacking glucose for 10 min. Glucose re-addition experiments were conducted by adding the requisite amount of glucose (2%) directly to cultures at the times indicated in the text. Cycloheximide was added to growing cultures at a concentration of 100 μg/mL as described [11] but was not present in the lysis buffer (pH 7.5) since a pH of greater than 7 is known to degrade cycloheximide. Control experiments found no difference in the results when cycloheximide was included in the lysis buffer or when it was not.

### AU analyses

Flag eluted samples (350 μL) were subjected to AU analysis by using A_230_ absorption or a fluorescence detection system (AU-FDS) [44] to detect GFP-fusion proteins [11, 15]. All analytical ultracentrifugation experiments were conducted at 20°C and at a rotor speed of 15,000 rpm. Twelve μm Spin60 centrifuge cells were used for the AUC experiments. At least 150 scans for AU-FDS experiments were obtained and at least 75 scans for AU- A_230_ analysis. Data were analyzed by SEDFIT software as described previously [11]. Stoichiometric analyses were done on samples that were split and run at the same time on two different AUC instruments, one for AU-FDS analysis and one for AU-A_230_ or AU-A_260_ analysis as described in the text.
